# Complete Genome Sequence of the Probiotic Strain *Lactobacillus casei* (Formerly *Lactobacillus paracasei*) LOCK919

**DOI:** 10.1128/genomeA.00758-13

**Published:** 2013-09-26

**Authors:** Anna Koryszewska-Bagińska, Tamara Aleksandrzak-Piekarczyk, Jacek Bardowski

**Affiliations:** Institute of Biochemistry and Biophysics, Polish Academy of Sciences, Warsaw, Poland

## Abstract

*Lactobacillus casei* is usually regarded as a bacterium that lives naturally in the human intestinal tract, where it can contribute to host health and well-being. We describe here the complete genome sequence of *L. casei* LOCK919, a strain with probiotic properties isolated from child feces. The genome consists of a 3.11-Mb chromosome and a 29,768-bp plasmid.

## GENOME ANNOUNCEMENT

*Lactobacillus casei* LOCK919 (formerly *Lactobacillus paracasei* LOCK919; Polish patent no. 209986), obtained from the Pure Culture Collection of Technical University (Lódz, Poland), was originally isolated from a fecal sample collected from healthy 5-year-old boy. In this study, LOCK919 strain was attributed to the species *L. casei* on the basis of 16S rRNA, *rpoA*, and *pheS* gene analyses (>99% sequence identity with that of *L. casei*) (1).

Based on earlier studies, it has been shown that strain LOCK919 is safe and meets the criteria for a probiotic strain, as its antagonistic activity against pathogenic bacteria and resistance to the stress encountered during gastrointestinal passage have been demonstrated (2, 3). Other documented properties relate to its ability to adhere to the Caco-2 epithelial cell line (4) and induction of Th1-type cytokines and regulatory transforming growth factor beta-1 (TGF-β_1_) in the blood cell cultures of children with allergies (5).

The genomic DNA was extracted by using the Genomic Mini purification kit (A&A Biotechnology). Two different types of libraries were prepared, a shotgun library of sheared genomic DNA and an 8-kb paired-end library, which then were sequenced on the GS FLX sequencer platform (Roche). Sequence assembly was carried out using the Newbler Assembler version 2.4 software (Roche). Automatic annotation of genes was generated using the RAST annotation server (http:/rast.nmpdr.org/) (6) with subsequent manual inspection. tRNAs were predicted using tRNAscan-SE (7).

The complete genome of *L. casei* LOCK919 is composed of a circular 3,113,601-bp chromosome and a 29,768-bp plasmid named pLOCK919, with mean G+C contents of 46.2% and 43.92%, respectively. There are 3,092 coding sequences (CDSs), 60 tRNAs, and 5 rRNA operons in the chromosome and 32 coding sequences in the plasmid.

The analysis obtained from the RAST server revealed 350 subsystems existing in LOCK919 and reported the absence of a subsystem feature for photosynthesis, which is observed in all 6 completely sequenced genomes of this species (8–13). The whole-genome sequence of LOCK919 has proven its safety, as no known pathogenic genes were identified.

Comparative genome analysis showed that the genome of LOCK919 is the largest one among the fully sequenced genomes of this species (8–13) and exhibits the greatest similarity to *L. casei* strain Zhang (13). Compared with other fully sequenced strains of *L. casei*, LOCK919 contains an increased number of genes distributed in categories of carbohydrates and phages or prophages. Indeed, one incomplete and 4 questionable phages were identified by the PHAST (phage search tool) server (14). Also, a reduced number of genes distributed in the category of cell wall and capsule was observed for the LOCK919 strain. As anticipated, the annotated genome of *L. casei* LOCK919 revealed the presence of factors relevant to the colonization of and persistence in the human gut, including proteins with putative roles in the adhesion to host structural factors. Additionally, several genes distributed over the plasmid pLOCK919 nucleotide sequence may be involved in the adherence to host cells.

### Nucleotide sequence accession numbers.

The genome information for the chromosome and the plasmid of *L. casei* LOCK919 has been deposited in the GenBank database with accession no. CP005486 and CP005487.
